# Aberrant Wnt/Beta-Catenin Pathway Activation in Dialysate-Induced Peritoneal Fibrosis

**DOI:** 10.3389/fphar.2017.00774

**Published:** 2017-10-30

**Authors:** Yuanyuan Guo, Lin Sun, Li Xiao, Rong Gou, Yudong Fang, Yan Liang, Ruiqiang Wang, Ningjun Li, Fuyou Liu, Lin Tang

**Affiliations:** ^1^Department of Nephrology, The First Affiliated Hospital of Zhengzhou University, Zhengzhou, China; ^2^Department of Nephrology, Second Xiangya Hospital, Central South University, Changsha, China; ^3^Department of Pharmacology & Toxicology, Medical College of Virginia Campus, Virginia Commonwealth University, Richmond, VA, United States

**Keywords:** Wnt/β-catenin signaling, peritoneal dialysis, mesothelial-mesenchymal transition, fibrosis, high glucose

## Abstract

Peritoneal dialysis (PD)-associated peritoneal fibrosis is a chronic progress which induces ultrafiltration failure. It remains a challenge to prevent the progression of PD-associated fibrosis in clinic practice. Wnt/β-catenin pathway plays important role in many severe fibrotic diseases, here we investigated its contribution to the development of peritoneal damage. We isolated mesothelial cells (MC) from the effluent of PD patients and found that the expressions of Wnt1, Wnt5a, β-catenin, and LEF1 were increased in patients with more than 1-year PD compared with patients who just started with PD (<1 month). The elevated expressions of Wnts and β-catenin were accompanied with changes in the expressions of E-cadherin, α-SMA, COL-I, and FN mRNA and proteins, which are known related to mesothelial-mesenchymal transition (MMT). In addition, treatment with high glucose significantly increased the expression of Wnt1, Wnt5a, β-catenin, and LEF1 as well as the expression of α-SMA, COL-I, and FN in human peritoneal mesothelial cells (HPMC), whereas the expression of E-cadherin was reduced. Dickkopf-1 (DKK-1) is an endogenous inhibitor of Wnt/β-catenin signaling. Overexpression of DKK1 transgene significantly decreased the expression of β-catenin and attenuated the process of MMT as indicated by the decreased expression of α-SMA, COL-I, and FN and the increased expression of E-cadherin. Furthermore, TGF-β1 treatment significantly activated the Wnt/β-catenin pathway in HPMCs, while DKK1 blocked the TGF-β1-induced Wnt signaling activation and significantly inhibited the process of MMT. These data suggest that the canonical Wnt/β-catenin pathway plays an important role in the MMT and fibrosis induced by PD.

## Introduction

Peritoneal dialysis (PD) is an alternative treatment to hemodialysis and commonly used for end-stage renal disease. During PD, the peritoneal membrane (PM), composed of a continuous monolayer mesothelial cells (MC), acts as a permeable barrier in the progress of ultrafiltration and diffusion. In this process, long-term exposure to bioincompatible PD solution containing high concentration of glucose and glucose degradation products (GDP) could impair the PM, resulting in the denudation of the MCs, submesothelial fibrosis and angiogenesis, and finally causes the ultrafiltration failure and discontinuation of PD (Schwenger et al., 2006; Kaneko et al., 2007). MCs, a specific cell type that shares some characteristics with epithelial cells, could transformed into myofibroblasts under certain conditions, which is so-called mesothelial-mesenchymal transition (MMT; Rynne-Vidal et al., 2017). MMT is the main factor in favor of the peritoneal dysfunction related to PD (Yanez-Mo et al., 2003; Liu et al., 2015), and High Glucose (HG) itself is proved to induce the MMT process by modulating complex gene expression (Xu et al., 2003; Ksiazek et al., 2007; Kim et al., 2008; Yu et al., 2009). Thus, unraveling the mechanism of MMT is an urgent need for the prevention of peritoneal dysfunction during PD. Studies have revealed that multiple factors play pathogenetic roles in the MMT transition of PD dysfunction, among which the TGF-β1 may be the most important one (Kang et al., 1999; Margetts et al., 2001; Loureiro et al., 2011, 2013). Considering the fact that the TGF-β1 is physiologically pleiotropic, non-discriminate targeting of TGF-β1 signaling may induce undesirable side effects. Therefore, to prevent the peritoneal dysfunction with limited secondary consequences, it is better to identify new signals which take part in the PD-associated fibrosis.

**Figure 1:**
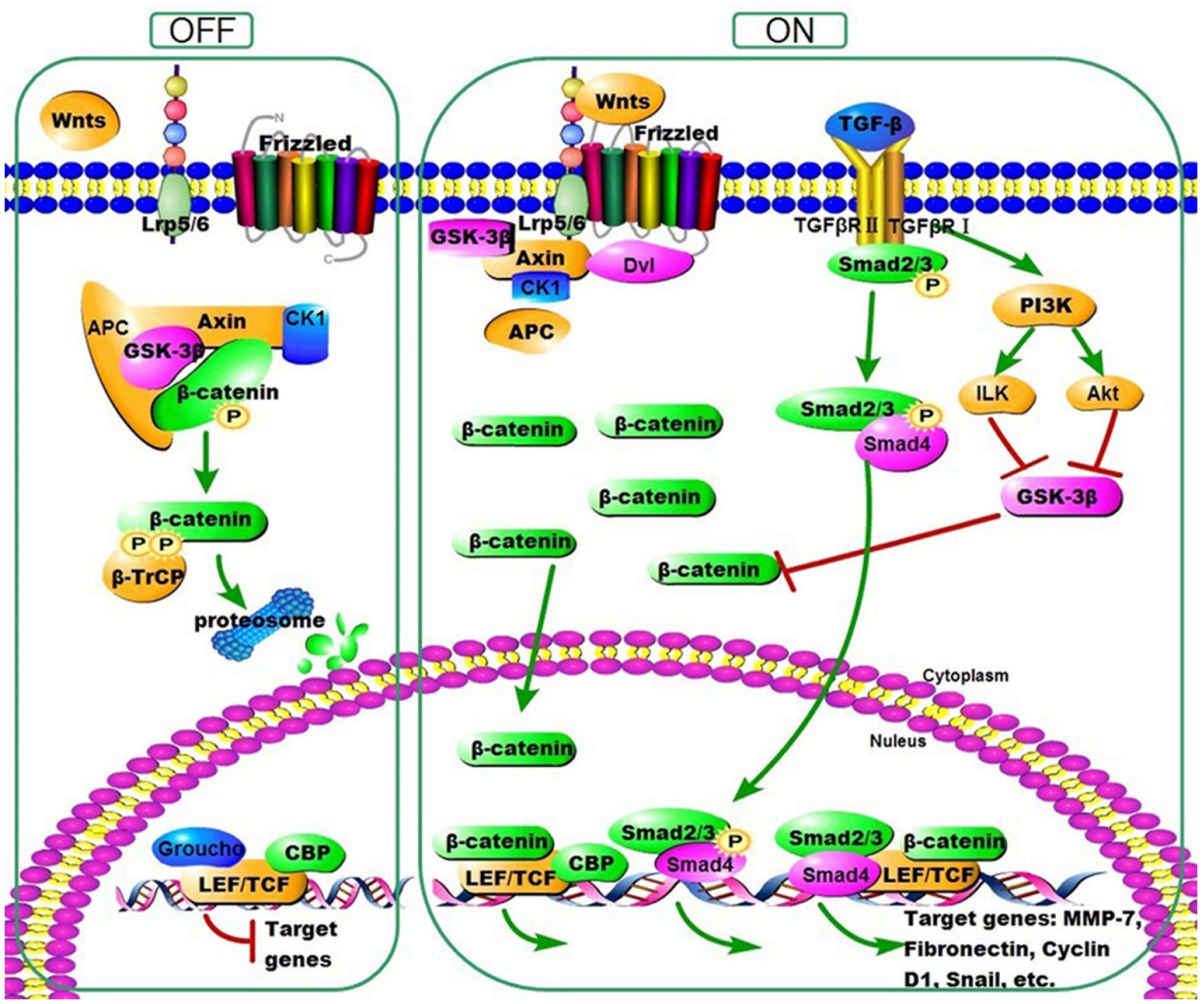
Overview of Wnt/β-Catenin Signaling. Without Wnt ligands, cytoplasmic β-catenin is phosphorylated by a destruction complex. Phosphorylated β-catenin is then ubiquitinated and destructed by the proteasome ultimately. Once activated, Wnt proteins bind to their receptors and induce a series of downstream signaling events which lead to the disassembly of the destruction complex, resulting in dephosphorylation of β-catenin. Accumulated β-catenin then translocates into the nucleus, where they bind to T cell factor (TCF)/Lymphoid enhancer-binding factor (LEF) to stimulate the transcription of Wnt target genes, including fibrosis-related gene expression, such as Fibronectin, Matrix Metallo Proteinases-7 (MMP-7), Twist, and Snail. In addition, Wnt/β-Catenin and TGF-β signaling converge at the promoter, where Smads, β-Catenin, and LEF/TCF form a complex to coregulate specific genes expression. β-catenin is further stabilized in the cytoplasm by the action of TGF-β to signal through phosphatidyl inositol 3-kinase (PI3K).

Wnt family proteins comprise various secreted lipid-modified signaling glycoproteins that deliver signals into a cell through the cell surface receptors (Wodarz and Nusse, 1998; Chien et al., 2009). At least three Wnt signaling pathways have been characterized, including Wnt/β-catenin signaling pathway (Clevers, 2006; Macdonald et al., 2007; MacDonald et al., 2009; Coombs et al., 2008; Staal et al., 2008). In the canonical response, binding of Wnt ligands to Frizzled/Low Density Lipoprotein Receptor Related Proteins 5/6 (LRP5/6) receptor complexes initiates a series of molecular events, resulting in the cytoplasmic stabilization and translocation of β-catenin to the nucleus. In the nucleus, β-catenin stimulates the transcription of Wnt target genes, including fibrosis-related gene expression, such as Fibronectin, Matrix Metallo Proteinases-7 (MMP-7), Plasminogen activator inhibitor-1 (PAI-1), Twist and Snail via binding to T cell factor (TCF)/Lymphoid enhancer binding factor (LEF). While under resting condition, in the absence of Wnt ligands, cytoplasmic β-catenin is phosphorylated by a destruction complex which followed by ubiquitination and finally destructed by the proteasomes (Figure 1). It has been reported that several secreted protein families play the antagonistic role in Wnt/β-catenin signaling pathway. Taking the secreted Dickkopf (DKK) family (DKK1–4), exemplified by DKK1, as an example, it could induce the LRP5/6 internalization and inactivation, and subsequently inhibit the formation of the receptor complex (Clevers and Nusse, 2012). Many studies showed that the Wnt/β-catenin pathway plays important role in epithelial-mesenchymal transition (EMT) and severe fibrotic diseases, such as pulmonary fibrosis, liver fibrosis, and renal fibrosis (Chilosi et al., 2003; Cheng et al., 2008; Konigshoff et al., 2008; He et al., 2009; Kim et al., 2011; Tao et al., 2016; Zhu et al., 2016). Furthermore, a recent study showed that intraperitoneal injection with 4.25% glucose PD fluid induced thicker peritoneum and activation of β-catenin signaling in mice (Ji et al., 2017).

In this work, we investigated the role of Wnt/β-catenin signaling in long-term PD-treated patients and in human peritoenal mesothelial cells (HPMC) exposed to HG and TGF-β1, and its possible relationship with MMT changes.

## Materials and methods

### Obtaining the HPMCs

Twenty four patients undergoing continuous ambulatory PD (CAPD) were enrolled, including 12 patients receiving CAPD for more than 1 year, and the other 12 patients receiving CAPD for less than 1 month. Before the first episode of peritonitis occurred, HPMCs were harvested from the effluents of PD patients as described (Yanez-Mo et al., 2003; Xiao et al., 2015b). Briefly, HPMCs were collected through a low-speed (1,500 r.p.m.) centrifugation. HPMCs were cultured with DMEM that contained L-glutamine and supplemented with 15% FBS, 0.5 μg/ml insulin, 5 μg/ml transferrin, and 1 μg/ml hydrocortisone in the humidified air with 5% CO_2_ at 37°C after three time wash. On the next day, non-adherent cells were removed with fresh culture medium added. After the culture of two passages (about 15 days), the collected HPMCs were used for various experiments. The purity of effluents-derived MCs was determined by flow cytometry. The study was approved by the Institutional Review Board and Ethics Committee of The Second Xiangya Hospital, Central South University, China. All participants thoroughly read and then signed the informed consent form.

### Culture of MCs line

Human peritoneal mesothelial cell line HMrSV5 was a gift from Dr. Pierre Ronco (Tenon Hospital, Paris), and cultured with the DMEM/F12 medium (Invitrogen) containing 10% fetal bovine serum (glucose concentration, 5.6 mM, as control). After reaching 80% confluence, the cells were divided as different groups with different culture media and used the serum-free medium for culture. In the experimental group, DMEM/F12 medium was supplemented with D-glucose (Kanto Chemical Co., Inc., Tokyo, Japan) at various concentrations (high glucose, 30, 60, 90 mM) or with D-mannitol (90 mM), or with recombinant TGF-β1 (R&D Systems, Minneapolis, MN) at gradient (2.5, 5, and 10 ng/ml; Xu et al., 2003; Ksiazek et al., 2007; Kim et al., 2008; Yu et al., 2009). In various combinations, the cells were transfected with pcDNA3.0-Flag-DKK1 plasmid or empty pcDNA3.0 plasmid.

### Real-time-PCR (RT-PCR)

To detect the expression of various genes, total RNA (500 ng) was used for reverse transcription and the cDNA was synthesized as described previously (Lin et al., 2002). The expression of Wnts, β-catenin, LEF1, DKK1, E-cadherin, α-SMA, COL-I, and FN in mRNA level were analyzed by TaqMan gene expression assays kit with Real-time PCR system 7300 (Applied Biosystems, Life Technologies). The relative expressions of genes were normalized to β-actin. The sequences of the primers were designed using the Primer Premier 5.0: Wnt1 sense: 5′-GTGGGGTATTGTGAACGTAGC-3′ and antisense: 5′-CGGATTTTGGCGTATCAGAC-3′, Wnt2 Sense: 5′-GTTCTTGAAACAAGAGTGCAAGTG-3′ and antisense: 5′-CCCATTGTACTTCCTCCAGAGATA-3′, Wnt2b sense: 5′-TCTTGTCTACTTTGACAACTCTCCA-3′ and antisense: 5′-GTCTGTTCCTTTTGATGTCTTGC-3′, Wnt3 sense: 5′-ACTTCCTCAAGGACAAGTATGACAG-3′ and antisense: 5′-GGGGAGTTCTCGTAGTAGACCAG-3′, Wnt3a sense: 5′-CCCCACTCGGATACTTCTTACTC-3′ and antisense: 5′-GCATGATCTCCACGTAGTTCC-3′, Wnt4 sense: 5′-CACTGAAGGAGAAGTTTGATGGT-3′ and antisense: 5′-AGTACACCAGGTCCTCATCTGTG-3′, Wnt5a sense: 5′-CAATGTCTTCCAAGTTCTTCCTAGT-3′ and antisense: 5′-CTTCTGACATCTGAACAGGGTTATT-3′, Wnt7b sense: 5′-CACCTGCTGAAGGAGAAGTACAAC-3′ and antisense: 5′-CTTCTCAATGTACACCAGGTCTGT-3′, Wnt8a sense: 5′-GAGGCGGAACTGATCTTTTTAG-3′ and antisense: 5′-CTGTTCTGTAGGCACTCACGAC-3′, Wnt10a sense: 5′-GGTGCTCCTGTTCTTCCTACTG-3′ and antisense: 5′-CTGGCAATGTTAGGCACACTG-3′, β-catenin sense: 5′-CCTTCCTGGGCATGGAGTC-3′ and antisense: 5′-GAGGAGCAATGATCTTGATCTTC-3′, DKK1 sense: 5′-AGTGTGTACCAAGCATAGGAGAAAA-3′ and antisense: 5′-TTAGTGTCTCTGACAAGTGTGAAGC-3′, LEF1 sense: 5′-ATGTCCAGGTTTTCCCATCATA-3′ and antisense: 5′-AGTGTGGGGATGTTCCTGTTT-3′, E-cadherin sense: 5′-GTCACTGACACCAACGATAATCCT-3′, and antisense: 5′-TTTCAGTGTGGTGATTACGACGTTA-3′ α-SMA sense: 5′-CCTCCCTTGAGAAGAGTTACGA-3′ and antisense: 5′-GATGCTGTTGTAGGTGGTTTCA-3′, Fibronectin sense: 5′-TGGAGGAAGCCGAGGTTT-3′ and antisense: 5′-CAGCGGTTTGCGATGGTA-3′, COL-I sense: 5′-AGAGTGGAGAGTACTGGATTGACC-3′ and antisense: 5′-GGTTCTTGCTGATGTACCAGTTC-3′,β-actin sense: 5′-CCTTCCTGGGCATGGAGTC-3′ and antisense: 5′-GAGGAGCAATGATCTTGATCTTC-3′. All experiments were carried out in duplicate.

### Western blotting analyses

Protein expression was assessed by western blot analysis as we described previously (Xiao et al., 2015b). In brief, protein (50 μg) samples were resolved by 10% SDS–PAGE and transferred to PVDF membranes, then probed with various primary antibodies against E-cadherin (1:500), α-SMA (1:500), COL-I (1:500) (Santa Cruz Biotechnology) and β-catenin (1:500) (Sigma-Aldrich, St Louis, MO, USA). After incubating with an appropriate secondary antibody, the protein expression was detected by western blot analysis, as described previously (He et al., 2009). β-actin (Santa Cruz Biotechnology, 1:1,000) was used as an internal control.

### Immunofluorescence analysis

The HPMCs were cultured on coverslips, and washed with PBS twice. Then the cells were fixed in 4% paraformaldehyde for 20 min, and then permeabilized with 0.1% Triton X-100. Next, 5% BSA was used to block the samples for 1.5 h at room temperature and then the following primary antibodies were used: anti-E-cadherin (1:100 dilution), anti-α-SMA (1:100) and COL-I (1:100) from Santa Cruz Biotechnology; anti-β-catenin (1:100) from Sigma. The antibodies were diluted in 5% BSA and cells were incubated with them overnight at 4°C, followed by incubation with FITC-labeled secondary antibody (Santa Cruz Biotechnology) at 22°C for 2 h. Nuclei was counterstained with 4,6-diamidino-2-phenylindole (DAPI). A UV fluorescence microscope (Leica, Shanghai, China; Magnification objective, ×40.) was used to visualize the immunostained cells and the intensity was analyzed with the image analysis software (Path, QC, Logene Biological Medical Engineering).

### Transfection of plasmid pcDNA3.0-FLAG-DKK1

pcDNA3.0-FLAG-DKK1 was a generous present from Prof. Youhua Liu (University of Pittsburgh, America). HMrSV5 cells were seeded in 6-well plates previously. After 12 h, the pcDNA3.0-FLAG-DKK1 or pcDNA3.0 empty vector as control was mixed with Lipofectamine (2 μg/μl, METAFECTENETM PRO) in serum-free Opti-MEMI at room temperature for 30 min according to the manufacturer's instructions and then the mixture was incubated with the HMrSV5 cells at a humidified 5% (v/v) CO2 atmosphere at 37°C for 5 h. To reduce the toxicity of Lipofectamine, cells were washed with fresh complete medium after transfection.

### Statistical analyses

The data were presented as mean ± s.e.m. Data were examined using ANOVA (Fisher's protected least significant difference test) for the multiple comparisons. Multiple comparison between the groups was performed using S-N-K method. Two-tailed Student's *t*-test was applied for the two group comparison. Normality test has been done before doing ANOVA and *t*-test. *P* < 0.05 was considered as statistically significant.

## Results

### Wnts and β-catenin were upregulated in the HPMCs from the effluent of long-term PD patients

**Figure 2:**
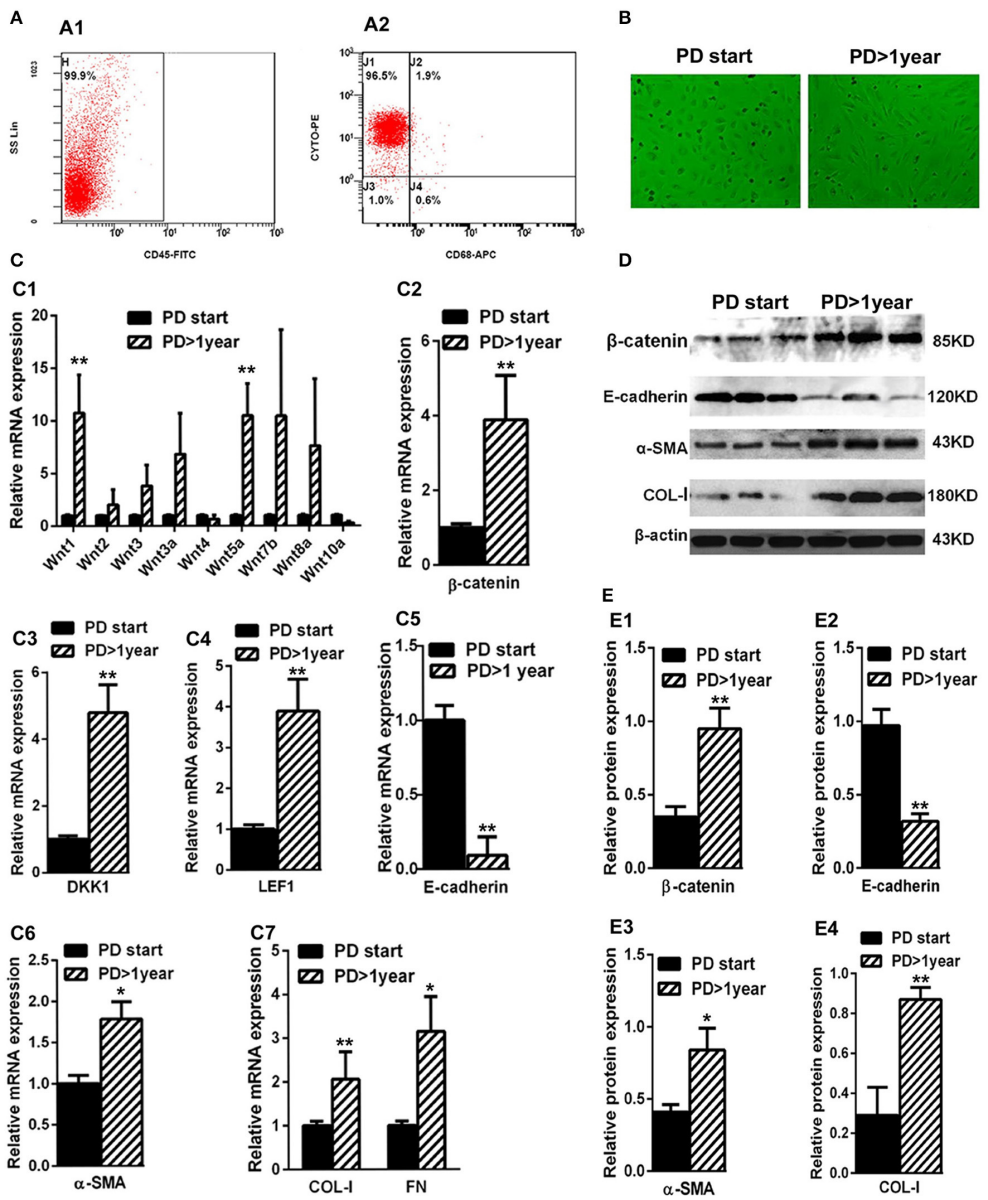
Wnts, β-catenin, DKK1, LEF1, E-cadherin, α-SMA, COL-I, and FN expression in the MCsisolated from effluent of PD patients. **(A)** The identification of MCs isolated from effluent of PD patients with flow cytometry (cytokeratin^+^CD45^−^CD68^−^). **(B)** The shape transformation of HPMC. **(C,C1–C7)** The relative mRNA level of Wnt1, Wnt2, Wnt3, Wnt3a, Wnt4, Wnt5a, Wnt7b,Wnt8a, Wnt10a, β-catenin, DKK1, LEF1, E-cadherin, α-SMA, COL-I, and FN in PD start group and PD > 1 year group. **(D)** The expression of β-catenin, E-cadherin, α-SMA, COL-I and β-actin in PD start group and PD>1 year group analyzed by western blotting. **(E,E1–E4)** The density of relative bands depicted of β-catenin, E-cadherin, α-SMA, COL-I by western blotting. Values are the mean ± s.e.m, *n* = 3, ^**^P < 0.01, ^*^P < 0.05 vs. control (PD-start group).

The average duration of dialysis in the PD> 1 year group (19.32 ± 8.73 months) was significantly longer than the PD-start group (0.53 ± 0.26 months). The other clinical characteristics, including gender, age, BMI, and renal failure causes, showed no significant differences. The expressions of proteins related to the Wnts pathway (Wnts, β-catenin, DKK1, and LEF1) were detected in HPMCs isolated from PD patients. As shown in Figure 2A, the percentage of peritoneal MCs (characterized as cytokeratin^+^CD45^−^CD68^−^) was accounted for nearly 96.3% (99.9 × 96.5%) in the cells isolated and cultivated from the effluent of PD patients in both PD > 1 year and PD start groups. The shape of the cells was different in the two groups. Specifically, the cells isolated from the PD-start group had round like shape, but most of the cells from the PD>1 year patients were spindle-shape (Figure 2B).

Real-Time-PCR data showed that the expressions of several Wnts, especially Wnt1 and Wnt5a, were significantly upregulated in the PD > 1 year group compared with the PD-start group (Figure 2C1). The expression of β-catenin (Figure 2C2) in the PD > 1 year group was upregulated nearly three-fold compared with the PD-start group. Similarly, the mRNA level of DKK1 (Figure 2C3) and LEF1 (Figure 2C4) were also increased in the PD >1 year group. Furthermore, the different expressions of β-catenin were further validated by western blot analyses (Figures 2D,E1).

We also found that the expressions of MMT-related genes and proteins were also altered. While the expression of E-cadherin was significantly decreased in the PD > 1 year group in both mRNA and protein levels (Figures 1D, 2C5,E2), the expressions of mesenchymal markers, including α-SMA, COL-I and FN, increased compared with the PD-start group (Figures 2C6,C7,D,E3,E4). The association between the changes of Wnts pathway and mesenchymal markers suggested that Wnt/β-catenin signaling may be involved in the MMT progression during PD.

### High-glucose treatment elevated the expression of wnts as well as β-catenin in HPMCs

**Figure 3:**
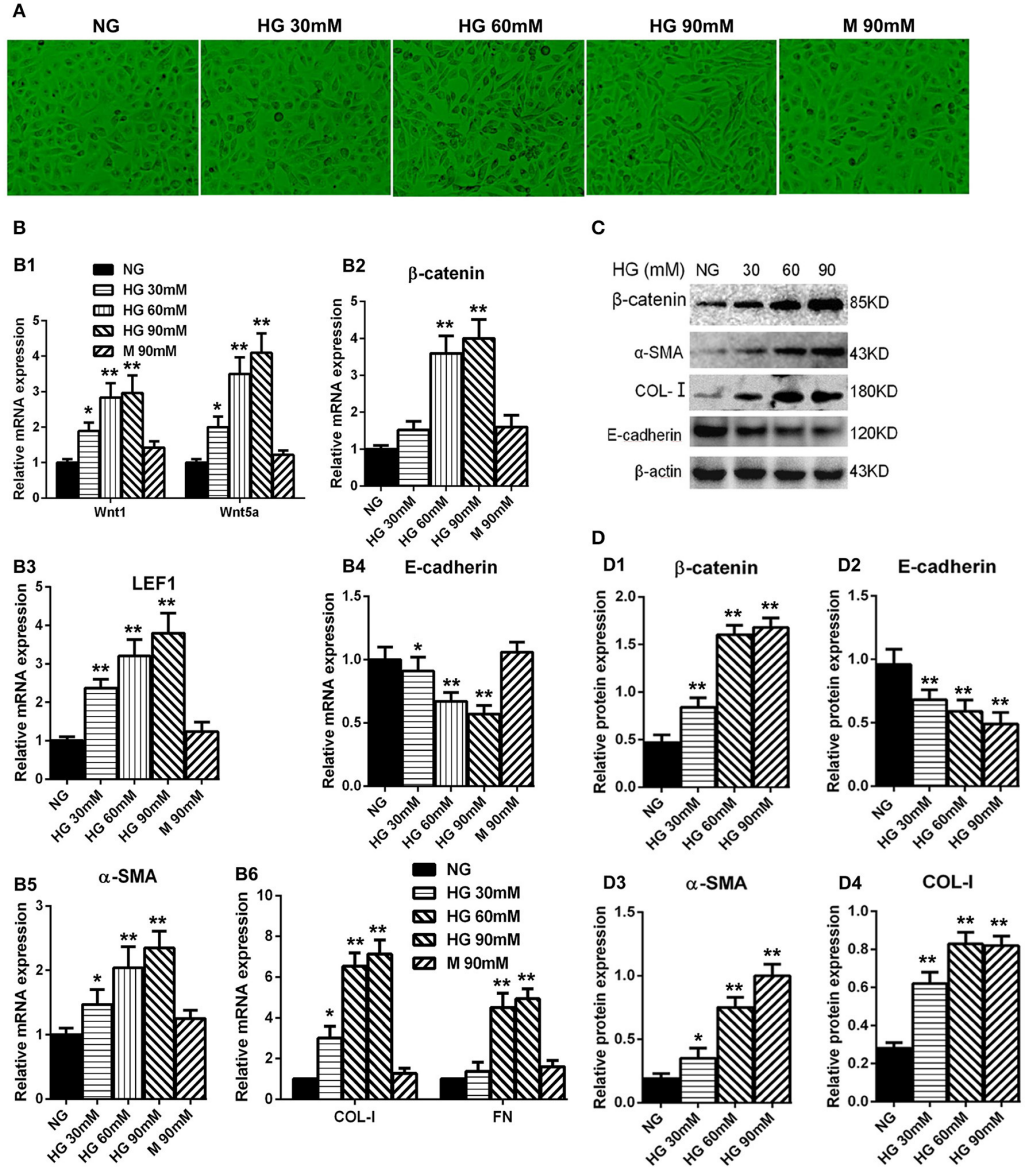
Analysis of Wnt related genes expression in cultured HPMCs under different glucose condition. **(A)** The shape transformation of HPMCs. **(B,B1–B6)** The relative mRNA level of Wnt1, Wnt5a, β-catenin, LEF1, E-cadherin, α-SMA, COL-I, and FN analyzed by real-time PCR. **(C)** The protein level of β-catenin, E-cadherin, α-SMA, COL-I analyzed by western blotting. **(D,D1–D4)** The density of relative bands depicted of β-catenin, E-cadherin, α-SMA, COL-I by western blotting. Values are the mean ± s.e.m., *n* = 3, ^**^P < 0.01, ^*^P < 0.05 vs. control (5.6 mM glucose).

We performed the experiment to examine the high-glucose effect on the HPMCs. The HPMCs were cultured with different concentrations of glucose for 24 h, including normal glucose group (NG: 5.6 mM of glucose), high glucose groups (30, 60, 90 mM of glucose), and osmotic control group (90 mM of mannitol). Interestingly, HPMCs became longer and had fibroblast-like shape when cultured with the increased glucose concentration (Figure 3A). The expression of Wnt1 and Wnt5a in the high-glucose groups were remarkably up-regulated compared with the control groups in a dose-dependent manner (Figure 3B1). Consistent with the changes of Wnts, the expressions of β-catenin (Figure 3B2) and LEF1 (Figure 3B3) were also significantly upregulated in the high-glucose groups. Furthermore, mesenchymal markers, including α-SMA, COL-I, and FN, had paralleled up-regulation in the high-glucose groups (Figures 3B5,B6), while the expression of E-cadherin was decreased with the glucose treatment in a concentration-dependent manner (Figure 3B4). The similar results were observed in protein level (Figures 3C,D). Taken together, we concluded that high glucose treatment could activate the Wnts pathway and may promote the MMT process.

### Overexpression of DKK1 transgene blocked MMT transformation in HPMCs treated with high-glucose

**Figure 4:**
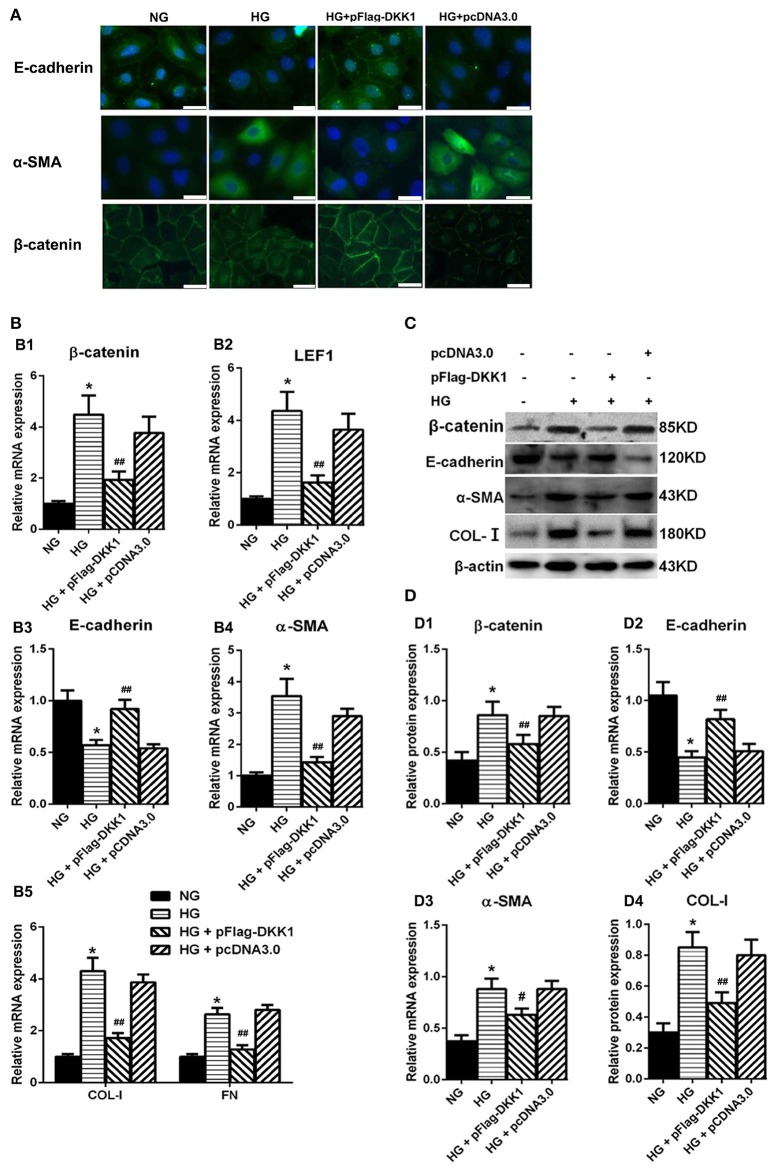
Overexpression of DKK1 Blocked MMT Transformation in HPMCs Treated with High-Glucose. **(A)** Immunofluorescence microscopy of E-cadherin (green), α-SMA (green), and β-catenin (green) in HPMCs in negative control (NG), HG (High-Glucose), HG + pFlag-DDK1 and HG + pcDNA3.0 group. Nucleus is stained with DAPI: blue). Magnification objective, ×40. Scale bar: 25μm. **(B,B1–B5)** The relative mRNA level of β-catenin, LEF1, E-cadherin, α-SMA, COL-I, and FN analyzed by real-time PCR. **(C)** The protein level of β-catenin, E-cadherin, α-SMA, COL-I analyzed by western blotting. **(D,D1–D4)** The density of relative bands depicted of β-catenin, E-cadherin, α-SMA, COL-I by western blotting. Values are the mean ± s.e.m., *n* = 3, ^*^P < 0.01 vs. control (NG), ^##^*P* < 0.01, ^#^*P* < 0.05 vs. HG.

The plasmids expressing DKK1 was transfected into HPMCs and then exposed to 60 mM glucose. The immunofluorescence staining data showed that β-catenin was significantly increased in the nuclear region of HPMCs stimulated with high glucose, whereas the signal intensity was restored in cells transfected with DKK1. The expression of α-SMA had similar trend (Figure 4A). On the other hand, over-expression of DKK1 significantly reversed the HG-induced inhibition of E-cadherin expression (Figure 4A). Notably, there was no difference in the DKK1-negative control plamids-treated group compared with the high glucose group. Also, the RT-PCR data showed that overexpression of DKK1 reduced the increased expressions of β-catenin and LEF1 induced by high glucose, but the DKK1-negative group had no such change (Figure 4B1,B2). Besides, we found that transfection of DKK1 partially reversed the inhibitory effect of High Glucose on E-cadherin (Figure 4B3), in opposite to the results observed in the α-SMA, COL-I, and FN (Figures 4B4,B5). Western blot analysis showed the similar results (Figures 4C,D). These results indicated that DKK1 transgene could partially block the Wnt/β-catenin signaling, and reverse the MMT process induced by high glucose in HPMCs.

### TGF-β1 elevated the levels of wnts and enhanced the expression of β-catenin in HPMCs

**Figure 5:**
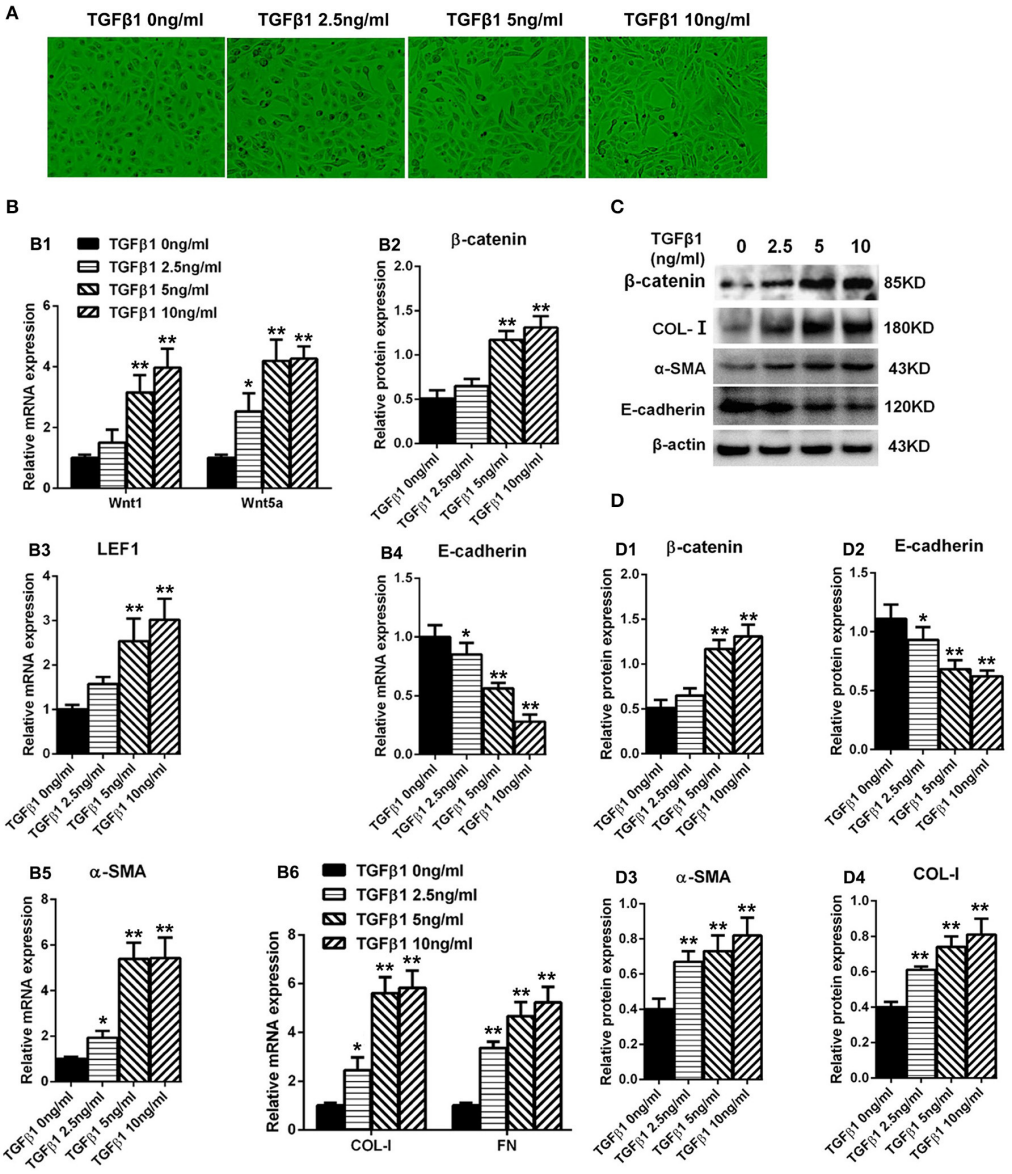
Expression of Wnt1, Wnt5a, β-catenin, LEF1, and epithelial-mesenchymal transition-related genes in HPMCs induced by TGF-β1. **(A)** The shape transformation of HPMCs with different concentration of TGF-β1 treatment. **(B,B1–B6)** The relative mRNA level of Wnt1, Wnt5a, β-catenin, LEF1, E-cadherin, α-SMA, COL-I, and FN analyzed by real-time PCR. **(C)** The protein level of β-catenin, E-cadherin, α-SMA, COL-I analyzed by western blotting. **(D,D1–D4)** The density of relative bands depicted of β-catenin, E-cadherin, α-SMA, COL-I by western blotting. Values are the mean ± s.e.m., *n* = 3, ^**^P < 0.01 vs. control (TGF-β10 ng/ml), ^*^P < 0.05 vs. control (TGF-β1 0 ng/ml).

The HPMCs turned into fibroblast-like form after cultured with TGF-β1 (Figure 5A). Real-Time PCR was performed to detect the expression of Wnt1 and Wnt5a in HPMCs incubated with TGF-β1 for 24 h, and showed that both Wnt1 and Wnt5a expressions were increased in a dose-dependent manner (Figure 5B1). Consistent with Wnts, the expression of β-catenin (Figure 5B2) and LEF1 (Figure 5B3) was also upregulated significantly and the similar trend was observed in α-SMA, COL-I, and FN (Figures 5B5,B6). Besides, the expression of E-cadherin was decreased by TGF-β1 in a concentration-dependent manner (Figure 5B4).The similar results were observed in protein level (Figures 5C,D).

### Overexpression of DKK1 transgene blocked MMT transformation in HPMCs treated with TGF-β1

**Figure 6:**
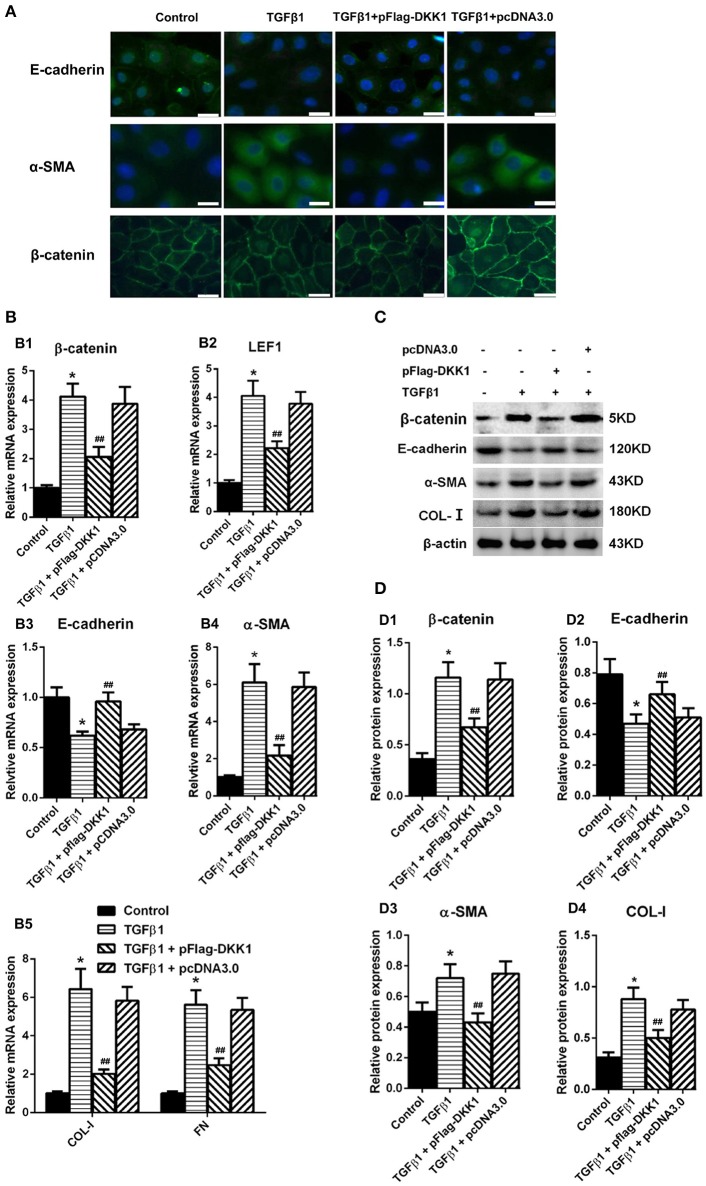
Overexpression of DKK1 decreases the expression of β-catenin and reverses MMT-related genes and proteins expression in HPMCs following treatment with TGF-β1. **(A)** Immunofluorescence microscopy of E-cadherin (green), α-SMA (green), and β-catenin (green) in HPMCs in control, TGF-β1, TGF-β1+pFlag-DDK1, and TGF-β1+pcDNA3.0 group. Nucleus is stained with DAPI: blue). Magnification objective, ×40. Scale bar: 25 μm. **(B,B1–B5)** The relative mRNA level of β-catenin, LEF1, E-cadherin, α-SMA, COL-I, and FN analyzed by real-time PCR. **(C)** The protein level of β-catenin, E-cadherin, α-SMA, COL-I analyzed by western blotting. **(D,D1–D4)** The density of relative bands depicted of β-catenin, E-cadherin, α-SMA, COL-I by western blotting. Values are the mean ± s.e.m., *n* = 3, ^*^P < 0.01 vs. control, ^##^*P* < 0.01, ^#^*P* < 0.05 vs. TGF-β1.

HPMCs treated with transfection of DKK1 or mock-transfection of DKK1 were exposed to TGF-β1 and showed that the expression of β-catenin was significantly increased in the nuclear region in cells with mock transfection, and that the signal intensity was restored in cells transfected with DKK1 (Figure 6A). Similar results were observed in α-SMA (Figure 6A). On the other hand, over-expression of DKK1 significantly reversed the inhibitory effects of E-cadherin induced by TGF-β1 (Figure 6A). There was no difference in the mock-transfected cells treated with TGF-β1. The RT-PCR showed that overexpression of DKK1 significantly reversed the β-catenin (Figure 6B1) and LEF1 (Figure 6B2) expression, whereas no such change was observed in mock-transfected DKK1 cells. In addition, the TGF-β1-induced inhibition of the E-cadherin was blocked in cells transfected with DKK1 (Figure 6B3), and that the opposite results were observed for α-SMA, COL-I, and FN expression (Figures 6B4,B5). Similar results were observed in protein level (Figures 6C,D). These results suggested that overexpression of DKK1 transgene partially blocks the Wnt/β-catenin signaling pathway and reverse the TGF-β1-induced MMT process in HPMCs.

## Discussion

Our data revealed that the main family members of the Wnt/β-catenin signaling pathway, such as Wnts, β-catenin, and LEF1, were significantly increased in HPMCs isolated from the effluents of long-term PD patients. Those changes positively correlated with MMT and peritoneal fibrosis process. Likewise, the Wnt/β-catenin signaling pathway was activated by High Glucose or TGF-β1 treatment *in vitro*. Importantly, treatment of HPMCs with the Wnt/β-catenin signaling inhibitor DKK-1 led to the inhibition of High Glucose- or TGF-β1-induced MMT.

### MMT and PD-associated fibrosis

PD is a prior therapeutic strategy for patients with end-stage renal disease. The process uses the patient's peritoneum as a semipermeable membrane to clear wastes and extra fluid. The primary advantage of PD is greater patient mobility and autonomy because it does not require frequent visits to hospital or medical facility. However, continuously exposure to non-physiological peritoneal dialysis solution (PDS) will inevitably lead to peritoneal fibrosis, resulting in the ultrafiltration failure, and increased morbidity as well as mortality in PD patients (Devuyst et al., 2002). The establishment of peritoneal fibrosis has been associated with MMT of the peritoneal MCs monolayer. Concurrent with a loss of epithelial cell adhesion and cytoskeletal components, cells undergoing MMT acquire the expression of mesenchymal components and manifest a migratory phenotype. Yanez-Mo et al. demonstrated mesothelial cells from dialysate effluents showed a progressive loss of epithelial characteristics and acquired a fibroblast-like phenotype, and previous studies have demonstrated that high glucose peritoneal dialysates can damage MCs both *in vivo* and *in vitro* (Jorres et al., 1994; Yanez-Mo et al., 2003). Many cytokines and pathways have been found to take part in this process, and TGF-β1 is generally considered as a master molecule. In addition, Notch signaling, PI3K/AKT signaling, Toll-like receptor induced signaling pathway, and others, have drawn attention in recent years (Zhu et al., 2010; Xiao et al., 2015a; Choi et al., 2017). Furthermore, the role of non-coding RNA has been studied, and a few microRNAs, like microRNA-129-5p, have been identified to be related with fibrosis of the peritoneum (Xiao et al., 2015b). Nevertheless, to date, the exact mechanisms of peritoneal damage during PD still remains unclear, and no effective therapeutic strategies is found. In our study, we demonstrated that Wnt/β-catenin signaling pathway is elevated during PDF-induced peritoneal fibrosis, reminding it maybe a potential therapeutic target in the treatment of PD-associated dysfunction.

### Wnt/β-catenin pathway and fibrosis

The detailed functions of Wnt signaling, reported to be associated with biological development and multiple physiological activities, are still unclear and remain to be further clarified. The most important and canonical one, Wnt/β-catenin pathway, is well understood currently. In embryonic development and organ morphogenesis, the Wnt/β-catenin signaling pathway has played a vital role. So it is reasonable that dysregulation of this pathway in adult may be linked to fibroblast biology and fibrosis (Clevers and Nusse, 2012). Fibrosis is an important response to protect tissue from further injury. The latest evidence suggests that Wnts are dysregulated and could mediate the EMT and fibrosis processes (Guo et al., 2012; Enzo et al., 2015). The biologic effects of Wnt signaling depend on the specific Wnt ligand, receptor complex and cell type. A specific Wnt protein may not activate β-catenin signaling in one tissue context, but is active in a different setting. Dai C et al. reported the upregulation of Wnt1 and activation of β-catenin in podocytes from patient samples of diabetic nephropathy and focal segmental glomerulosclerosis. In addition, He et al. showed that the majority of Wnt family and Fzd receptors were involved in the unilateral ureteral obstruction (UUO) model and would induce the progressive interstitial fibrosis and tubular atrophy (He et al., 2009). Moreover, the obstruction could lead to a dramatic accumulation of β-catenin in the tubular epithelial cells, paralleled by an increase in the expression of Wnt/β-catenin target genes (He et al., 2009). Further, β-catenin plays an additional role as a component of adherens junctions through binding with various cadherins such as E-cadherin. The E-cadherin-β-catenin complex is associated with actin filaments, thus forming a dynamic link with the actin cytoskeleton. It's also very important for the maintenance of epithelial cell layers. E-cadherin has a peculiar distribution in MCs, since it is expressed both in membrane and in cytoplasm. Unlike the signaling pool of β-catenin, it is thought that β-catenin binding to the adherent junction is highly stable. The role of β-catenin in Wnt family-induced signal transduction and cell adhesion may be mostly dependent on its phosphorylation at different sites (Heuberger and Birchmeier, 2010). Most importantly, the activation of β-catenin was recently observed in the peritoneum of mice which received intraperitoneal injection with PD fluid (Ji et al., 2017). Thus, we performed a comprehensive analysis of canonical Wnt signaling components in PD patients and HG-treated HPMCs. Our work showed that the expression of many members of Wnt family, especially Wnt1 and Wnt5a, and β-catenin, were increased in both MCs harvested from the effluents of PD patients or HPMCs cells treated with High Glucose. These changes were accompanied by decreased expression of E-cadherin and upregulated expression of COL-I and FN. These data indicate that Wnt/β-catenin signaling may have a significant role in the process of MMT and fibrosis in PD.

### Therapeutic strategies

Recent studies have demonstrated that TGF-β1-induced MMT plays a central role in the pathogenesis of peritoneal fibrosis in PD. However, because TGF-β1 could induce the immune and inflammatory responses, the direct inhibition of the TGF-β1 signaling may cause immune suppression. Other signaling pathways, such as Wnt/β-catenin, may be the alternative candidate to prevent the progression of PD-associated fibrosis. Recently, the inhibition of the Wnt signaling pathway has been reported to regulate the fibrotic responses in non-peritoneal tissues (Lin et al., 2010). Several methods have been used to block the Wnt/β-catenin signaling pathway including the soluble receptors, inhibitors, siRNA, and transcription competitor or inhibitors (Surendran et al., 2005; Hao et al., 2011; He et al., 2011; Kim et al., 2011; Matsuyama et al., 2014). Dickkopf (DKK), the antagonistic inhibitor of the Wnt signaling pathway, can isolate the LRP6 co-receptor from participating in activating the Wnt signaling pathway (Mao et al., 2002). When DKK was administered locally in a naked plasmid vector or systemically in an adenoviral vector for gene therapy, the interfering of the canonical Wnt pathway restrained the renal interstitial fibrosis, hepatic cell activation and liver fibrosis (Cheng et al., 2008; He et al., 2009). Here, overexpression of DKK-1 transgene significantly decreased β-catenin accumulation, reduced the expression of Wnt/β-catenin target genes and suppressed the type I collagen and fibronectin producing induced by HG and TGF-β1, suggesting that Wnt/β-catenin signaling antagonism may represent a useful strategy for the treatment of PD-associated fibrosis.

### Cross-talk between Wnt/β-catenin and TGF-β signaling

Interestingly, the crosstalk between TGF-β1 and Wnt pathways has been identified for a long time (Medici et al., 2008; Figure 1). And the cooperation of TGF-β1 and Wnt/β-catenin signaling in the process of fibrosis has been confirmed recently (Scheraga and Thannickal, 2014; Lu et al., 2016). Wang et al. reported that the Wnt/β-catenin signaling pathway was activated and their target genes were upregulated in TGF-β1-induced podocyte injury and proteinuria *in vitro* and *in vivo*, and this process was interrupted by Wnt antagonist DKK1, suggesting that Wnt/β-catenin pathway is the downstream of TGF-β1(Wang et al., 2011). Furthermore, it has been found that TGF-β-induced Wnt/β-catenin signaling pathway was transcribed by Smad3 and p38MAPK pathways in human dermal fibroblasts, and that p-β-catenin/p-Smad2 complexes were observed in alveolar epithelial cells during the fibrotic phase in the lung tissue from IPF patients both *in vitro* and *in vivo* (Sato, 2006; Kim et al., 2009). Besides, knockdown of β-catenin with siRNA significantly reduced the TGF-β secretion in the lung tissue of bleomycin administered mice, and that loss and gain function of DKK1 impact the High Glucose-induced TGF-β secretion, suggesting that the β-catenin may induce the expression of TGF-β (Lin et al., 2010; Kim et al., 2011). Our results have shown that TGF-β1 significantly upregulates the expression of Wnts, β-catenin, and the Wnt target genes, and that this process is inhibited by the delivery of DKK-1 transgene *in vitro*. These data indicate that Wnt/β-catenin signaling acts in a combinatorial manner with TGF-β signal in the progression of PD-related fibrosis.

In summary, our data revealed that the activation of Wnt/β-catenin signaling pathway mediated the MMT process and peritoneal fibrosis, and that the suppression of Wnt/β-catenin signaling pathway restrained the development of peritoneal fibrosis. These findings may provide information for developing novel therapeutic strategies associated with Wnt/β-catenin signaling pathway for peritoneal fibrosis in patients undergoing PD.

## Ethics statements

This study was carried out in accordance with the recommendations of Institutional Review Board and Ethics Committee of Second Xiangya Hospital, Central South University with written informed consent from all subjects. All subjects gave written informed consent in accordance with the Declaration of Helsinki. The protocol was approved by the Institutional Review Board and Ethics Committee of The Second Xiangya Hospital, Central South University.

## Author contributions

YG, FL, and LT conceived and designed the study. RG, YF, RW, and YL performed the experiments. All authors analyzed data. YG and LX wrote the paper. LS, FL, LT, and NL reviewed and edited the manuscript. All authors read and approved the manuscript.

### Conflict of interest statement

The authors declare that the research was conducted in the absence of any commercial or financial relationships that could be construed as a potential conflict of interest.
